# A comparison of emulsion stability for different OSA-modified waxy maize emulsifiers: Granules, dissolved starch, and non-solvent precipitates

**DOI:** 10.1371/journal.pone.0210690

**Published:** 2019-02-06

**Authors:** Hisfazilah Saari, Marie Wahlgren, Marilyn Rayner, Malin Sjöö, María Matos

**Affiliations:** 1 Department of Food Technology, Engineering and Nutrition, Lund University, Lund, Sweden; 2 Department of Chemical and Environmental Engineering, University of Oviedo, Julian Claveria 8, Oviedo, Spain; Shanghai Institutes for Biological Sciences, CHINA

## Abstract

This work investigates the stability of emulsions prepared by using octenyl succinic anhydride (OSA)-modified waxy maize starch in the form of granules, dissolved starch, and non-solvent precipitated starch as Pickering emulsion stabilisers. The aim of this study was to investigate the effects of different forms of starches on the stability of emulsion using light microscopy, light scattering, and static multiple light scattering. All starch samples were hydrophobically modified with 3% (w/w) n-octenyl succinyl anhydride (OSA). Starch polymer solutions were prepared by dissolving OSA- modified starch in water in an autoclave at 140°C. Non-solvent precipitates were obtained through ethanol precipitation of dissolved waxy maize. The stability of the oil/water emulsions were different for the three forms of starches used. The granule-based emulsions were unstable, with only a small proportion of the granules adsorbed onto oil droplets, as viewed under a light microscope. The emulsions were observed to cream after 2 hours. The dissolved starch and non-solvent precipitate-based emulsions were stable towards creaming for months, and they had almost 100% emulsifying index (EI = 1) by visual observation and EI ~ 0.9 by multiple light scattering measurements. The results from light microscopy and multiple light scattering measurements indicated the occurrence of coalescence for all three types of emulsions. The coalescence was fastest within days for the granule stabilised system while it was slower both for the dissolved starch and non-solvent precipitate-based emulsions. The latter demonstrated the least degree of coalescence over time. Thus, it was concluded that differences in starch particle size and molecular structure influenced the emulsion droplet size and stability. A decreased particle size correlates to a decrease in droplet size, thus increasing stabilisation against creaming. However, stability towards coalescence was low for the large granules but was best for the non-solvent precipitate starch indicating that there is a window of optimal particle size for stability. Thus, best emulsifying properties were obtained with the non-solvent precipitates (~ 120 nm particle size) where the emulsions remained stable after one year of storage. In conclusion, this study illustrated the potentiality of non-solvent precipitated starch as emulsion stabilizers.

## Introduction

Emulsions are widely used in formulations consisting of two immiscible liquids dispersed in one another in the form of small droplets. Since emulsions are thermodynamically unstable, emulsifiers or stabilisers are required to stabilize the droplets to prevent rapid re-coalescing. The emulsion droplet stabilization is often achieved through the addition of surfactants or emulsifiers which decrease the interfacial tension between the phases and increase the steric hindrances and/or electrostatic repulsion between the droplets, thereby increasing the stability of the emulsion [1, 2]. Traditionally, surfactants and polymers including natural polymers such as polysaccharides and proteins are used to stabilise emulsions. However, recently, particle-based emulsifiers piqued the interest of researchers as it has a wide range of technological applications [3–10]. Particle-based emulsifiers are known as Pickering emulsions which were first reported by Pickering and Ramsden, where, they can be obtained using different types of particles such as silica, latex, clay (synthetic/inorganic) [11, 12], and a range of bio-based particles [13, 14].

Many researchers concluded that larger solid particles (10 nm–5 μm) create a physical barrier between droplets, hence, produce a high desorption energy and adsorb irreversibly to the interface to a higher degree than other emulsifiers such as surfactants [2, 15]. Pickering emulsions have been observed to have better stability compared to other emulsions towards coalescence and Ostwald ripening [2]. However, their large size decelerates the kinetics of adsorption, thus, increases the number of particles (weight per volume of oil) needed for emulsification compared to the traditional emulsifiers. Such differences between particles and other stabilisers are strongly dependent on size, where it has been observed that nanoparticles around 1–5 nm diameter display similar behaviour to globular proteins due to a comparable size and desorption energy [2]. However, soluble polymers and particles vary significantly when subjected to adsorption to the water/oil interface. The structure of a particle remains unchanged while polymers obtain considerable flexibility leading to changes in the structure when adsorbed to the water/oil interface. Therefore, non-solvent precipitated starch that have been shown to produced structures in the nano-size range will be tested for Pickering type stabilization in this study [16].

General characteristics of starches which are derived from a renewable resource include low toxicity and tasteless. These characteristics enable its formulation application in food, beverages, cosmetics, pharmaceuticals, paints, and coatings [9, 17, 18]. Starch in its natural state is found as granules which appear as solid, semi-crystalline particles in the size range of 1–100 μm. However, the granules can be dissolved to obtain starch in its molecular form by extensive heating combined with mechanical treatment or by using solvents such as dimethyl sulfoxide (DMSO). Both the physical forms of starch can be used as an emulsifier for oil in water emulsions. The utilization of both forms has been reported in a previous study where the emulsifying properties were compared [6]. In addition, the dissolved OSA-modified starch is known as a good emulsifier [19–24]. The emulsifying capacity of OSA starches are dependent on a range of properties, consisting of molecular weight (increased molecular weight improves stability but increases droplet size), rigidity of the hydrophilic parts of the polymer chain (increases steric stability) and higher amounts of amylopectin with low hydrodynamic radius (decreases droplet size) [17]. During the emulsification with OSA starches, a preferential adsorption of high molecular species on the emulsion droplets is present [22]. However, high shear rate emulsification has been shown to decrease the molecular weight of the starches [20].

Furthermore, it has been reported that some emulsions which were stabilised using starch granules indicated long-term stability [15, 25, 26], for example, particle-stabilised emulsion from quinoa with 1–2 μm size remained stable up to 2 years of storage [26]. The character of starch Pickering emulsion is dependent on the size, morphology, and hydrophobicity of the starch [27, 28], as well as the starch concentration [2, 15]. In general, smaller starch granules produce more stable emulsions with smaller droplet size [25, 26, 28].

In order to achieve adequate adsorption at the oil/water interface, both the starch granules and dissolved starch were made to be more hydrophobic. This technique is performed primarily using chemical modification with octenyl succinate anhydride [23]. Using the method, the effect on the emulsion droplet size was non-linear with a higher degree of OSA substitution. Where the level of modification used in the study, 3%, was estimated to be more than sufficient to stabilise emulsions which is also the limit for modification allowed within food applications under the E-number E1450 [20].

On the other hand, many researchers have been using waxy maize as a subject material and widely studied, making this starch type a popular source for comparison. Waxy maize has irregular and sharp edges, with graininess, pin-holes, and deep depressions on the surface of some granules [29, 30]. The granules can be categorised into two populations, granules in the size range between 1–7 μm and 15–20 μm [29, 31]. In a previous study, waxy maize was used as a sole emulsifier and was able to stabilise the emulsion with a mean droplet size (d4,3) of 47 μm and an emulsifying index (EI) of 0.37. However, the EI improved to 0.47 when the granule size was reduced to half the initial size post acid hydrolysis treatment [28].

Since small particle size is known to decrease the droplet size with constant parameters where in some cases it increases the stability, nano-sized particles could be of interest to produce Pickering emulsions. Nano-sized starch structures have been produced using non-solvent precipitation [32–35]. A recent study from our research group revealed that non-solvent precipitation yielded material in the sizes range between 10 nm -1 μm [16]. This non-solvent precipitate produced stable emulsions with an EI of 1. Hence, the aim of this study is to compare the long-term emulsion stability of emulsions stabilised by octenyl succinic anhydride (OSA)-modified waxy maize starch in three different physical forms, which are the intact granules (Pickering emulsions), dissolved starch (polymer stabilised emulsions), and non-solvent precipitated starch (stabilisation principle unknown).

## Materials and methods

### Materials

The native waxy maize starch used was supplied by Lyckeby Culinar, Sweden. The oil used was the Miglyol 812, a medium-chain triglyceride (MCT) oil with a density of 945 kg/m^3^ (Sasol Germany). While the water phase was a 5 mM phosphate buffer with 0.2 M NaCl with a density of 1009.6 kg/m^3^. The n-octenyl succinyl anhydride (OSA) was obtained from Trigon Chemie, Germany.

The three different starch emulsifiers utilised in this study include; Starch granules based emulsions (SGE); Dissolved starch-based emulsions (DSE); and non-solvent precipitated starch-based emulsions (NPSE). The preparation of these emulsifiers is described below. All starches were OSA-modified.

### Methods

#### OSA-Modification

The amount of OSA used was based on the dry weight of starch measured by an IR-balance (135°C, 5 min, duplicate). The starch was dispersed in 1.5 to 2 times the amount of distilled water which was added during stirring. The pH was maintained at 7.4 and 7.8 where it was adjusted using citric acid, 1M HCl or NaOH. Then, 3–4% n-octenyl succinyl anhydride (Trigon Chemie, Germany) was added in 4 portions with a 15 min delay between each portion. The automatic titration of 1M NaOH while stirring was set to maintain the pH close to 7.6. The titration step was terminated once the pH was stable for at least 15 min. The suspension was then centrifuged at 3000g for 10 min to recover the starch granules. The supernatant was discarded, and the starch was washed twice with water, once with citric acid (pH 4.5–5) and once more with water. The starch was frozen overnight and freeze dried.

The determination of OSA level was carried out in a duplicate for modified and control samples. First, 2.5g of starch (dry weight) was wetted with a few drops of ethanol prior to the addition of 25ml 0.1M hydrochloric acid. The suspension was stirred for 30 minutes. Next, the suspension was centrifuged at 3000g for 10 min, where the supernatant discarded at the end. Pellet containing starch was washed with 25ml ethanol once, and with distilled water twice prior to dissolving it in 150ml distilled water and heated in a hot bath at 90°C for 10 min. The solution was rapidly cooled to 25°C using an ice bath, and the suspension was titrated while stirring with 0.1M NaOH until pH 8.3 was reached. The volume of NaOH used was recorded for the calculation of % OSA degree as below:

$$Degree\;of\;OSA\;modification\;(\%)=\frac{(Vsample-V\;control)\times M\times 210\times 100}{W}$$

Where V is the volume of NaOH required for titration (ml), M is the molarity of NaOH (0.1M), 210 is the molecular weight (g/mol) of octenyl succinate anhydride group (OSA) and W is the dry weight of sample used.

#### Preparation of starch granules (SG)

Starch granules which were obtained from the supplier Lyckeby Starch were modified to increase the hydrophobicity using 3% of OSA based on dry weight of starch following the method described above. Following OSA modification, the wet samples were put into a freezer overnight and freeze-dried (Hetosic, Denmark). The starting temperature was set at -20°C and was set to reach 20°C with an increase in temperature by 1°C/min over 4–5 days. The degree of OSA modification for starch granules was determined to be 3.1%

#### Preparation of dissolved starch solutions (DS)

The starting material for the dissolved starch was the OSA-modified starch granules prepared as described above. The OSA-modification was also the same as above. The OSA-modified starch granules were dissolved by adapting the dissolution methods reported by Perez-Rea et al. using an autoclave [36]. This method has previously been shown to completely dissolve starch without any substantial degradation of the dissolved molecules. A sample of OSA-modified waxy maize starch (4 g dry weight) was dispersed in 200 ml milliQ water and heated with a high-pressure laboratory autoclave coupled with a magnetic stirrer and a temperature control unit (WRX 2000, Withernm, Germany). The heating was carried out for 20 min at 140°C. Prior to heating, the system was flushed with nitrogen gas for 5 min to prevent oxidative degradation of the starch. The starch suspension was gradually heated from room temperature to 140°C for almost 14 min, maintained at 140°C for 20 min, and then cooled by immersing the autoclave cylinder in an ice bath. Following autoclave dissolution, the dissolved starch solution was diluted to 8 mg/mL from the initial concentration of 20 mg/ml before being used in emulsions. The methodology and optimization processes of dissolved starch preparation was taken from a previous study [16]

#### Preparation of non-solvent precipitated starch (NPS)

Non-OSA modified starch was dissolved in the autoclave as described in the previous section. Non-solvent precipitated starch (NPS) was obtained by directly mixing ethanol into dissolved starch (8 mg/ml concentration) at a ratio of 1:1 (starch solution: ethanol). The precipitated starch was collected after centrifugation (2000 g, 10 min). Following centrifugation, the solvent was removed and samples were left in the fume hood overnight to completely evaporate the residual ethanol in the precipitated particles. The precipitates were then subjected to OSA modification using the similar method employed in OSA starch granules preparation as described above. The degree of OSA modification for starch granules was determined at 2.03%. The procedure for preparing NPS was adapted from a previous work [16].

#### Preparation of emulsions

The emulsions produced consists of 5% (w/w) of oil/water emulsions containing an MCT oil for the dispersed phase and a phosphate buffer (5 mM, pH 7, 0.2 M NaCl) for a continuous phase, with 200 mg of starch emulsifier per ml of oil. The emulsions were produced by mixing 70 mg of modified granule/non-solvent precipitated (in dry form) or dissolved starch (in solution) into the 0.35 g oil/6.65 g buffer (total of 7 g emulsion composition). The emulsions prepared were mixed by vortexing for 10 s. Then, the suspensions were emulsified using a high-shear mixer (D-79282, Ystral, Germany) at 22000 rpm with a 6mm rotor diameter for 60 s. The stored emulsions that had not phase separated were observed after 24 hours, weekly for up to six weeks and also after a year.

### Characterisation of emulsions

#### Static light scattering

The particle size distribution of all emulsions was examined using static light scattering (Mastersizer 2000, Malvern Instruments, UK). A small quantity of sample was injected into the flow system connected to the pump circulating at 2000 rpm. The refractive index (RI) was set at 1.54 corresponding to starch [37], while for the continuous phase, it was set to 1.33 which is the RI of water. Generally, obscuration was restricted between 10% and 20% for the emulsions. However, due to the low amounts of samples, the obscuration was reduced to less than 5% when measuring emulsions based non-solvent precipitated/dissolved starch, since their size corresponds to a low degree of obstruction. The data, d4,3 and dv,0.5, refer to the sphere of equivalent volume and the median size of the system based on d4,3 distributions, respectively. The mean $\bar{\bar{d_{4,3}}}$ size of the systems based on the distribution was also determined. This incorporates all particles in the emulsions (emulsion droplets and free starch particles). At least two normally three or more different samples were analysed at each time point. All samples were measured in triplicates where the average and standard deviations of the $\bar{\bar{d_{4,3}}}$ mean was calculated. These average values were compared using two-sided student t-test and Anova analysis was calculated to measure difference between samples. Anova was made on the following series, difference over time for DSE, NPSE and the difference between GSE; DSE and NPSE at zero and 24 hour respectively.

#### Light microscope

Emulsions were further observed with an optical microscope (BX50, Olympus, Japan) equipped with a video camera. The samples were examined under Plan 2x, UMPlanFL 5x and 10x, and LMPlanFL 20x and 50x objectives (Olympus). One drop of sample was diluted with five drops of buffer solution. For particular samples, one drop of the diluted sample was placed uncovered on the glass plate for microscopic observation.

#### Stability of emulsions studied by static multiple light scattering

A sample size of 4 ml starch-based emulsions was prepared to study the emulsion stability during storage. Dispersions of OSA-modified starches (granule, dissolved starch, and non-solvent precipitated in buffer) and the MCT oil (oil in buffer) were also measured as a reference to compare with the emulsions. The stability study of the emulsions was evaluated using Turbiscan LabExpert (Formulation Inc., France) where it employs static multiple light scattering. The instrument operates by sending a light beam through a cylindrical glass cell containing 4 ml of the sample (12 mm height). The light source is an electroluminescent diode in the near infrared with a wavelength of 880 nm. Part of the incident light is then backscattered by the sample or transmitted through it and received by two sensors at different locations: the sensor that receives the transmitted light (TS) is located 180° from the incident radiation, while the sensor that receives the backscattered light (BS) is located 45° from the incident light. The TS and the BS were monitored as a function of time and cell height at 30°C. The TS and BS data were measured every 40 μm in % relative to standards (suspension of monodisperse spheres and silicone oil) as a function of the sample height (in mm).

The BS measurement is directly dependent on the particles’ mean diameter and on their volume fraction. The main instability phenomena observed in colloidal systems are: particle migration (i.e., local variation of the concentration of particles causes local variation of the transmission or backscattering level measured at the bottom and top of the sample) and increment in particle size (i.e., the global variation of the particle size causes global variation of the transmission or backscattering level measured in the middle of the sample) [6]. BS profiles build up a macroscopic fingerprint of the emulsions at a given time, providing useful information related to changes in droplet size and the appearance of a creaming layer or clarification front, which makes it possible to calculate the velocity [4].

The Turbiscan stability index (TSI) was obtained using Turbisoft software (2.0.0.33) using mathematical computing based on the sum of scan-to-scan difference of the intensity of lights (∑*h*|*scan*_*i*_−*scan*_*i*−1_) along the cell height. The sum of the variations (∑*i*) detected in the samples was calculated using the following equation:

$$TSI=\sum i\frac{\sum h|{scan}_{i}-{scan}_{i-1}|}{H}$$

where H is the total height of the cell. TSI sums all the variations detected in the samples in terms of size and/or concentration. The higher is the TSI, the higher the instability (no specific scale for this). Which means that the emulsions with the lowest TSI are more stable since there were no variations on the % of light transmitted or backscattered which correlates to no variations due to migration phenomena (e.g. sedimentation or creaming) or size variations (i.e., coalescence or flocculation).

#### Emulsifying index

The emulsifying index (EI) is the ratio between the volume of the emulsion layer and the volume of the whole sample. In the present study, the EI was calculated by measuring the height of the cell containing the emulsion layer (showing the backscattering peak) vs. the time from the Turbiscan profile. The emulsifying index was calculated as follows:

$$EI=\frac{Height\;of\;BS\;peak\;in\;the\;cell}{Total\;height\;of\;sample\;in\;the\;cell\;(12\;mm)}$$

## Results and discussion

Based on the described methodology, three different emulsion systems were investigated using waxy maize starch; OSA modified starch granules-based emulsions (SGE); OSA modified dissolved starch-based emulsions (DSE), and; OSA modified non-solvent precipitated starch-based emulsions (NPSE). SGE is a Pickering emulsion based on waxy maize granules that have a size of 5–18 μm with an average size around 15 μm [29] while DSE, a polymer stabilised emulsion that predominately contains amylopectin with a radius of gyration of around 250 nm [36]. Although the structure of the NPS is not yet fully understood, previous studies indicated that it contains a broad range of particles including larger micro-particles. However, the bulk of the material is small, with an average radius of gyration of 260 nm as measured by asymmetrical flow field-flow fractionation (AF4) [38].

**Fig 1:**
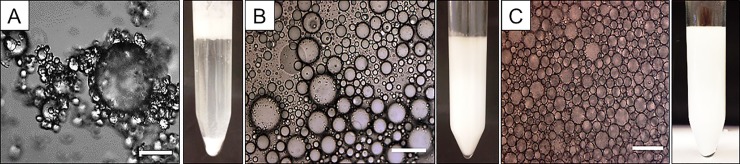
Emulsion, droplet size and macroscopic. Microscope images at 20x magnification, bar scale: 50 μm appearances of the emulsions on day 1 (A) SGE, (B) DSE, and (C) NPSE.

The macroscopic appearance of emulsions on day 1 is presented in Fig 1. Based on Fig 1, SGE was susceptible to gravitational separation, while DSE and NPSE yielded emulsions that were space filling. SGE exhibited creaming and sedimentation characteristics due to large oil droplets which cream while small droplets covered with starch granules sediment due to their higher density. The degree of separation can be followed using multiple light scattering which also determines EI. As for EI values, a significant difference was observed with highest values recorded for non-solvent precipitated (0.92), followed by dissolved starch (0.88), and the least for granule (0.47). Referring to Table 1, the EI value declined fastest for SGE and slowest for NPSE. For the former, an oil layer on top of the emulsion layer was observed after 24 hours. However, this was not observed for the other emulsions. In addition, based on the student’s t-test, a significant difference between the droplet sizes (Table 1) for the three emulsions was observed. These difference could explain the variation in the macroscopic behaviour of the emulsions where a higher tendency is present for the larger droplets of the SGE to cream.

**Table 1:** Emulsion properties; size measured by static light scattering in terms of mean volume diameter $\bar{d_{4,3}}$ (n = 3), median value of size distribution dv, 0.5 (n = 3), changes of backscattering over time, ΔBS (%), emulsifying index (EI n = 2, EI of 0.1 can also be expressed as 10% as stated in discussion), and Turbiscan stability index (TSI n = 2).

Emulsions	Days	$\bar{d_{4,3}}$ **(μm)**	****dv, 0.5(μm)****	****ΔBS (%)****	****EI****	****TSI****
****SGE****	0	45.8 ± 7.9**	26.0 ± 4.1	11.0	0.47±0.00	-
	1	74.0 ± 10*	56.4 ± 20.0	13.6	0.27±0.02	30.0±3.2
****DSE****	0	18.9 ± 5.3**	15.2 ± 0.9	27.0	0.88±0.02	-
	1	20.2 ± 3.1**	16.4 ± 1.0**	45.4	0.85±0.04**	7.5±7.0
	7	22.9 ± 6.0	17.2 ± 0.7	45.4	0.70±0.01*	14.2±2.5
	14	27.2 ± 6.0	17.7 ± 1.1	49.3	0.63±0.01*	16.2±0.6
	21	32.6 ± 4.2*	18.3 ± 0.9	50.2	0.58±0.00*	17.8±0.8
	28	42.8 ± 7.0*	24.8 ± 6.1	49.8	0.51±0.04*	18.8±1.5
	35	51.0 ± 4.0*	37.2 ± 4.0*	49.9	0.45±0.01*	19.5±1.6
	42	86.4 ± 0.6*	83.1 ± 1.0*	51.0	0.42±0.01*	19.8±1.3
	365	55.6 ± 10.2*	42.0 ± 19.0*	59.9	0.35±0.10	–
****NPSE****	0	10.6 ± 0.6**	9.3 ± 0.7**	39.4	0.92±0.01	-
	1	10.4 ± 0.8**	9.0 ± 0.9**	47.4	0.88±0.02*	5.7±7.0
	7	16.3 ± 4.2*	9.2 ± 1.0	49.6	0.76±0.01*	8.4±3.4
	14	19.1 ± 2.4	7.9 ± 1.5	53.0	0.75±0.01	12.5±0.6
	21	21.2 ± 5.0	7.3 ± 1.5	52.0	0.70±0.00*	14.9±0.4
	28	23.2 ± 9.6	6.7 ± 1.6	52.3	0.67±0.01*	15.8±0.4
	35	27.8 ± 8.0	5.7 ± 2.1	54.5	0.63±0.01*	16.5±0.6
	42	39.8 ± 10.3*	8.3 ± 3.5	53.4	0.63±0.01	17.0±1.0
	365	48.9 ± 10.7*	36.1 ± 16*	44.6*	0.47±0.00*	–
****MCT oil****	0	123.8 ± 15.4	117.2 ± 14.5	–	–	–

The values marked with * represent student’s t-test that are significantly different compared to time point with a 90% confidence interval.

Meanwhile the values marked with ** represent student t-test that are significantly different compared to the drop size of the other emulsions investigated at zero and 24 hours.

To further assure that there is difference between the samples, an Anova test was conducted to confirm the difference between the averages in the sample series. All Anova analyses verified that there are significantly different over time (NPSE and DSE emulsions) and between 3 groups of emulsions at zero and 24 hours with *p-value<0*.*05*. The statistical analysis is included in S1 Table.

The TSI-value (Table 1) is a measurement that incorporates all types of changes in a dispersion that affect the scattering profile. Therefore, the present study includes creaming, sedimentation and coalescence characteristics. The higher the TSI, the less stable is the emulsions. The TSI values are in concordance with the other results strongly indicating that NPSE is the most stable system while SGE is the most unstable emulsion. Besides, this behaviour also increases with time.

### Changes in droplet size over time as measured by static light scattering

**Fig 2:**
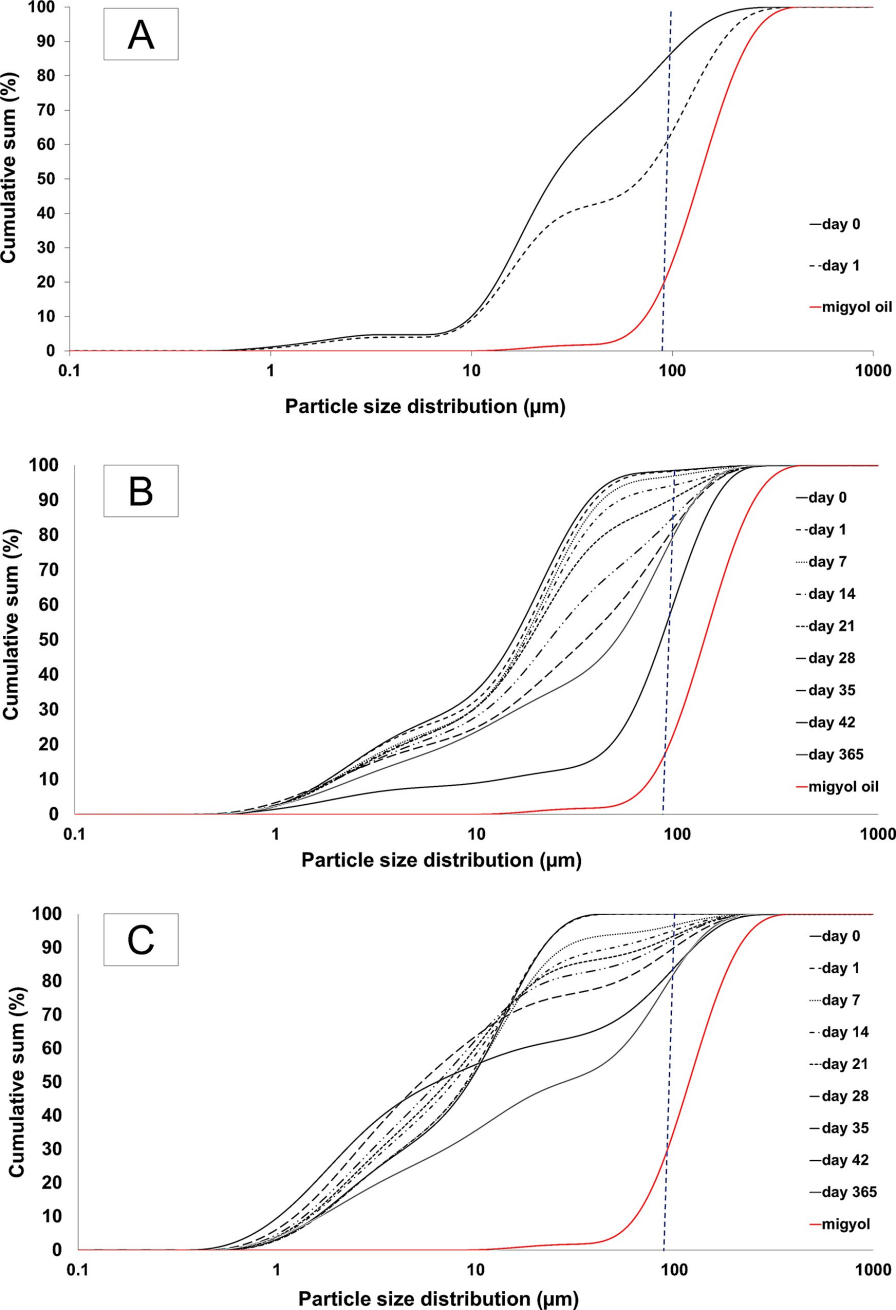
Size distribution of emulsions. Graph representing emulsions stabilised by (A) granules, SGE (day 0 to day 1), (B) dissolved starch, DSE (day 0 to day 365), and (C) precipitated starch, NPSE (day 0 to day 365).

In order to understand how the emulsions change with time, the changes in size distribution measured by static light scattering (Fig 2), including mean volume diameter $\bar{d_{3,4}}$ and median size dv, 0.5 (Table 1) were monitored during storage. The median size is, as mentioned in Table 1 differs from the average size. This variation indicated a skewed distribution, represented by a d_4,3_ distribution. Hence, the larger particles possess a significant influence on the average droplet size. The discussion that follows focuses primarily on the average size and not the median. The SGE system exhibited a significant increase in $\bar{d_{4,3}}$, from an initial value of 45 μm to 74 μm after the first 24 hours (Table 1), while no significant changes were reported for DSE and NPSE emulsions. However, as time progressed a gradual increase was measured for $\bar{d_{4,3}}$ in these systems. The gradual increase in $\bar{d_{4,3}}$ is most likely due to the coalescence of the emulsion droplets. On the other hand, the low solubility of MCT oil in water acts as the main destabilising mechanism is Ostwald ripening. The DSE system indicated a significant change in $\bar{d_{4,3}}$ between day 21 and 28, with a gradual increase over the interior time span. Besides, $\bar{d_{4,3}}$ for the NPSE also increased gradually, however, the changes between adjacent time points were not significant. But, over time it amounted to a significant change. Moreover, $\bar{d_{4,3}}$ and dv, 0.5 (μm) were quite similar for SGE and DSE systems suggesting that the particle size for these systems were close to being normally distributed while for the NPSE, these two values varied especially at later time points.

The change in particle size over time is illustrated in detail in the cumulative graph distributions (Fig 2 and S1 Fig). As a baseline reading, pure MCT oil was pumped through the particle size analyser for static light scattering and is displayed in Fig 2. Due to the mechanical treatments during analyses, oil droplets will be formed as free oil. The droplet size of MCT oil in buffer is around 90–140 μm, with an average size of 123 ± 15 μm (Table 1). Therefore, emulsions with droplet diameter above 90 μm were considered as large droplets obtained through coalescence or aggregates of droplets and were formed as free oil. As for SGE, the measurements were terminated after 24 hours because all parameters indicated a relatively rapid coalescence. It was also supported by visual observations and cumulative graph (Fig 2A) where free oil in the emulsion was present on day 1. The instability of the system could have been due to the low coverage of starch granules at the emulsion interface, supported by microscopic observations where large amounts of free starch were observed in the solution (Fig 1).

On the other hand, DSE possessed a nearly constant median droplet size (approximately 17 μm) until day 28, after which there was a significant increase to 85 μm. After 28 days, an increase in the number of droplets above 90 μm was observed (Fig 2B). The droplet size kept increasing after day 35, indicating that this could probably be the storage stability limit for this type of emulsion. Finally, the one-year data exhibited a high fraction of small particles. This change could be due to the formation of starch particles through retrogradation, as discussed below. The change in the curves indicated a slow coalescence of the droplets, but visual observation revealed the formation of free oil on top of the sample at the beginning of the study. However, oiling-off occurred to some degree during later time points. These findings postulate that emulsions stabilised by dissolved starch are more stable compared to the granule-stabilised emulsions with the presence of coalescence. The results are in concordance with the results from Sweedman et al (2014) who reported that waxy maize provides good long-term stability in emulsion formulations [17].

For NPSE, Fig 2C exhibited that emulsion droplets stabilised by NPSE had a median droplet size of approximately 6–9 μm up to 42 days. Thus, compared to DSE, NPSE emulsions did not record a significant change in the median droplet size during this time period. However, the number of droplets above 90 μm increased rapidly for NPSE than of DSE. It was observed as a gradual increase for the first 42 days as the cumulative sum for this fraction escalated from 0% to around 40%. Based on the observation from Fig 2C, the overall droplet size distribution did not shift towards larger droplets, instead, the fraction of droplets above 10 μm seemed to be coalescing. Hence, there is a clear difference in the coalescence pattern between NPSE and DSE emulsions. Since, NPSE has a wide population of sizes, from nano to micron-size [16], it is likely that this wide distribution also translates to a wider population of emulsion droplets stabilised by different entities of the precipitated starch. It can be speculated that some of these droplets are more stable than others towards coalescence, thus, making up the non-changing fraction of the emulsions. While droplets which are predominantly covered by different fractions of the non-solvent precipitated are more susceptible to coalescing. An additional explanation could refer to the creaming of the larger droplets on the top of the emulsions creating a higher density of droplets in this region to promote coalescence. Finally, at the end of the one-year storage, this emulsion still possessed a large fraction of small droplets. This strongly suggests stability towards droplet growth which is not affected by any chemical changes of the emulsion.

### Multiple light scatter measurements

**Fig 3:**
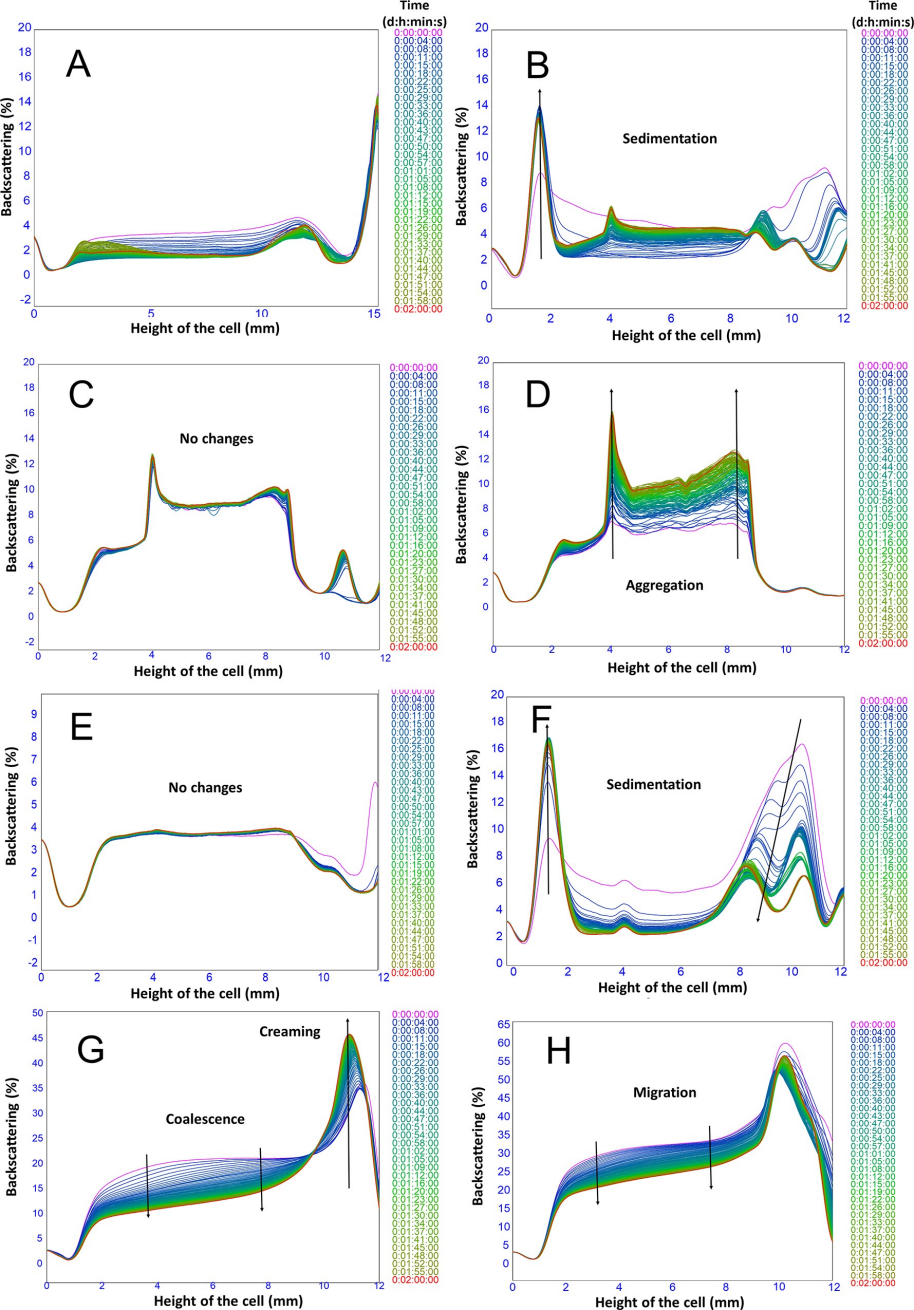
Turbiscan profiles of emulsions. Multiple light scattering profiles for starches in buffer: (A) MCT oil profile as reference (B) OSA–modified granules, (C) dissolved starch at day 0, (D) dissolved starched (DS) after 1 year, and (E) Non-solvent precipitated starch (NPS); (F) SGE, (G) DSE, (H) NPSE.

**Fig 4:**
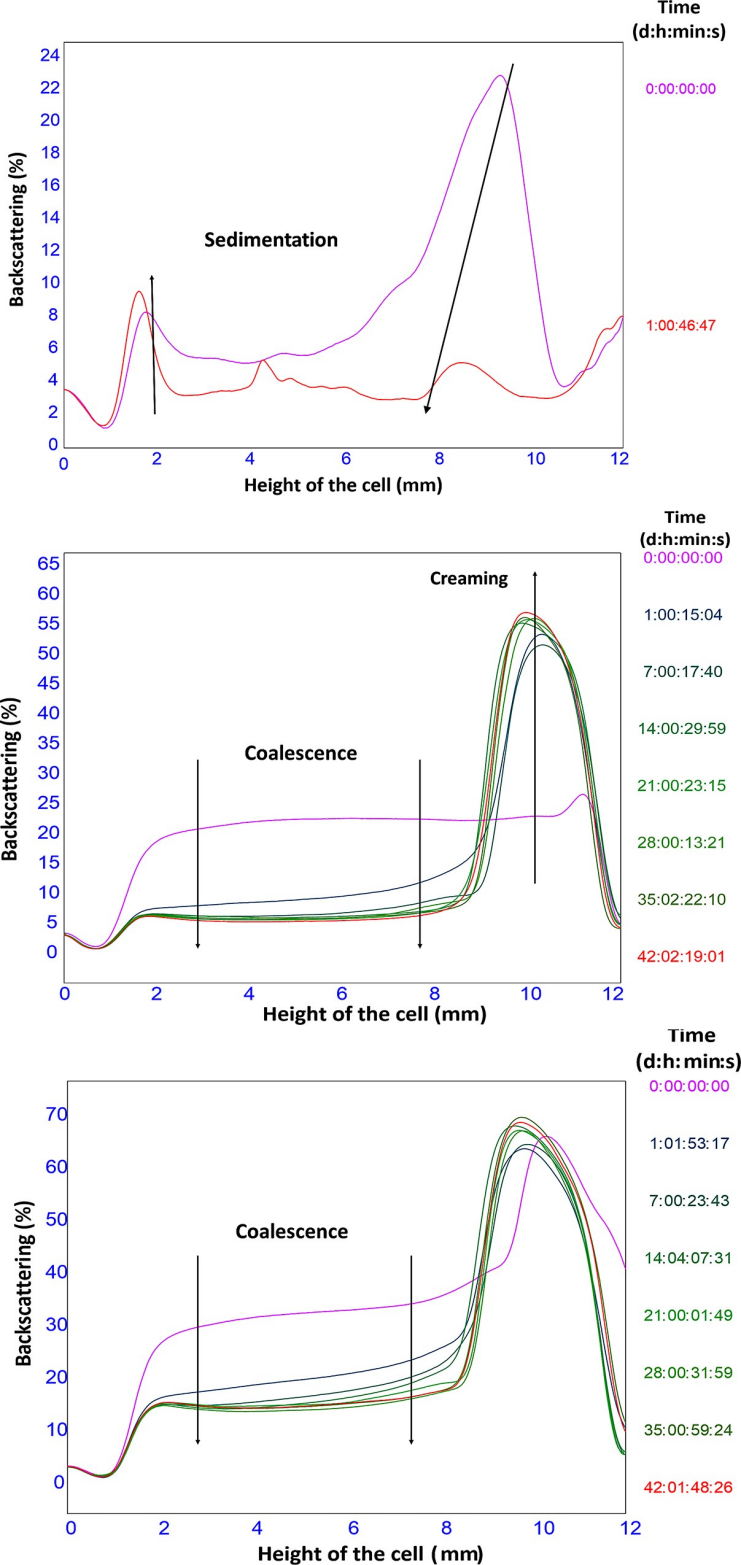
Turbiscan profiles of emulsions. Profiles representing starch-stabilised emulsions: (A) SGE after 1 day, (B) DSE, and (C) NPSE from 0 to 6 weeks.

The static multiple light scattering was measured using the Turbiscan LabExpert and the changes in backscattering during the first two hours is displayed in Fig 3, while the long-term stability investigations are illustrated in Fig 4.

To better understand the results for the emulsions, a multiple light scattering baseline data were also obtained for samples containing only the starch emulsifiers (Fig 3B–3E) and oil (Fig 3A). Based on Fig 3A, the backscattering is low over the whole height of the cell with a small “bump” at the top indicative of the free oil. The dispersed granules (Fig 3B) recorded high initial values in backscattering (in the range of 2–15%) at the bottom of the measurement cell (initial 1–2 mm), indicating that sedimentation had already occurred within this region of the samples.

When freshly dissolved OSA-modified starch was investigated (Fig 3C), backscattering decreased at the bottom of the cell (0–4 mm), indicating that clarification had occurred at the bottom. However, backscattering increased towards the middle of the cell (4–8 mm), most likely owing to the aggregation of the starch polymers. Initially, the system did not change with time. However, when the solution of dissolved starch was measured again after a year of storage (Fig 3D), it had a similar profile to that of the fresh sample, but, changes in backscattering was observed during the time of the measurement. The changes may have been due to continuous aggregation of starch in the solution, where the aggregates are large enough to scatter light. The large aggregates are the dissolved starch molecules which recrystallise in the solution, a process referred to as retrogradation. Retrogradation occurs during the cooling and storage stages, where both amylose and amylopectin crystalise in the solution [39, 40]. However, the rate of crystallisation for amylose is faster within a couple of hours or days, compared to amylopectin crystallisation where it occurs slowly after 30–40 days. Since waxy maize mostly contains an amylopectin fraction, the difference between the fresh and the stored sample could be attributed to the long-term crystallisation of the amylopectin fraction leading to long-term structural changes [39, 41, 42].

The backscattering profiles of samples containing only NPS exhibited that there were very small changes in the profile (Fig 3E). Since the particles obtained with non-solvent precipitation are small, they were subjected to Brownian motion and remained suspended without forming any clarification zones in the sample. In addition, NPS did not display any major changes over time compared to DS which was linked to crystallisation. The observation is suggestive of the non-solvent precipitation creating particles or starch molecules which differ in properties compared to DS.

On the other hand, in terms of stability, SGE was very unstable as displayed in the backscattering profiles (Figs 3E and 4A). Larger BS value (approximately 16%) was noted for SGE at the top of the cells at the beginning of the monitoring time resulted from the creaming of the emulsion droplets. While high initial BS values (in the range of 8–16%) were recorded at the bottom of the cell. In the latter case, it could have been due to sedimentation which may have resulted from the presence of free starch granules in the continuous phase which lasted until the end of the monitoring time. Meanwhile, an increase in backscattering in the middle part of the cell (4–8 mm) was measured probably due to the presence of either starch or emulsion sediment. Similar behaviour has been obtained with starch Pickering emulsions in previous studies [6]. On the other hand, the cream layer becomes thinner with time with the appearance of a free oil layer, which is another indication of the low stability of SGE (Fig 4A).

The BS profiles obtained for the DSE (Figs 3D and 4B) demonstrated large variation over time in the middle part of the cell as a result of clarification and creaming processes. Creaming was noticed at the top, resulting from droplet migration which was promoted by density differences between the oil droplets and the continuous phase causing a large change in backscattering from 27% to 51% (Table 1).

Additionally, the backscattering profiles of the NPSE (Figs 3H and 4C) exhibited a similar trend to that of DSE with similar variations in the middle of the cell. These variations were promoted by a clarification process that took place at the bottom/middle of the cell accompanied by simultaneous creaming at the top. However, the change in the middle of the cell related to coalescence was slower than that of DSE.

Based on the results above, a comparison between the three forms of starches can be discussed. The stability of the emulsion systems seemed to increase in the order of SGE << DSE < NPSE. This was true for stability towards both creaming and coalescence. The SGE showed clear phase separation and release of oil after 24 hours, while the emulsions stabilised by DSE were stable in this respect for 6 weeks and the NPSE were stable for at least one year.

Since all the particles studied originated from the same starting material, the size and morphology of the emulsifiers play a role in affecting the emulsion stability as it was reported in previous studies [43]. The low stability of starch granules was due to the particle size which was not small enough to create small emulsion droplets, indirectly affecting the creaming and coalescence properties of the emulsion. Also, since the affinity of the starch granules for oil/water interfaces is low, high amounts of free starch particles were observed in the samples.

Although the DS and NPS were in the same particle size range, the stability of the two emulsifiers varied from each other significantly. Based on previous studies, dissolved OSA waxy starch has been described as a good emulsifier, which was attributed to the adsorbed amylopectin inducing steric repulsion at the interface, as well as increased viscosity in the solution that to some extent hinders creaming [20, 21, 23, 44]. These previous observations are in line with the obtained results, however, a loss in stability was observed after 35 days due to slow retrogradation of the dissolved amylopectin. Retrogradation is a common phenomenon for dissolved starch [39, 41, 42] and is driven by the formation of starch double helixes that are further aggregated into ordered structures that lead to the formation of semi-crystalline arrays of these helixes [45]. Retrogradation can, in turn, lead to the formation of aggregates, hence, depleting the free available OSA starch to stabilise the emulsion.

The lack of aggregates observed in the Turbiscan profile for the NPS compared with the profile of the DS indicates that the NPS affects the tendency for any free amylopectin to later retrograde and form new aggregates in solution. This allows NPS to remain stable for a longer period of time than that of DS. In addition, NPSE which has a smaller initial droplet size than DSE indicates NPS consisting of smaller particles can cover a larger surface area creating a higher affinity to the surface. One other explanation could be that the particles do not have to be closely packed at the surface in order to stabilise the emulsion. This is because, based on previous observations on the Pickering type of emulsions, the stability can still be obtained below full surface coverage [8]. However, to assure this preferential adsorption of larger particles or aggregates of particles at the oil/water interface would be preferred. The transport of molecules and particles to an interface can be achieved through diffusion or convection, and when convection dominates, larger molecules will be preferentially adsorbed to the interface [46, 47]. The larger particles in an approximate size of 1μm are transported through convection, where the presence of these larger particles could help to stabilise the emulsion. When large particles have been adsorbed to the interface, it is very unlikely that they will desorb [2]. Thus, it is highly likely that the larger particles of NPS will be preferentially adsorbed and the droplets that have enough of these particles will be more stable than droplets covered by dissolved amylopectin only. This scenario could explain the coalescence profile for the NPSE in this study, where two fractions of droplets were present with the small droplets being very stable over time while the slightly larger droplets appeared to coalesce with time.

## Conclusions

The different sizes and shapes of the waxy maize-based emulsifiers (granules, dissolved starch, and precipitated starch) are capable of influencing the resulting emulsion droplet size and stability. The decrease in droplet size and the increase in stability towards coalescence and creaming were found to follow the order; SG (largest, least stable), DS, and NPS (smallest, most stable). The observed stability of the emulsions towards coalescence increased for the three OSA modified starches in the order; SGE<< DSE<< NPSE. In general, based on the droplet size, size distribution, microscopic images and Turbiscan stability, NPSE is the most stable followed by dissolved starch and granules. Therefore, in conclusion, this study revealed the high potentiality of non-solvent precipitated starch, NPS as emulsifiers.

## Supporting information

S1 Fig Emulsion droplet size and Micrograph. (TIF)

S1 Table Statistical analysis data. (DOCX)
