# Supplementary figures and images for: A comparison of emulsion stability for different OSA-modified waxy maize emulsifiers: Granules, dissolved starch, and non-solvent precipitates
Source: PLoS One. 2019 Feb 6;14(2):e0210690. doi: 10.1371/journal.pone.0210690 (PMC6364883; doi:10.1371/journal.pone.0210690)

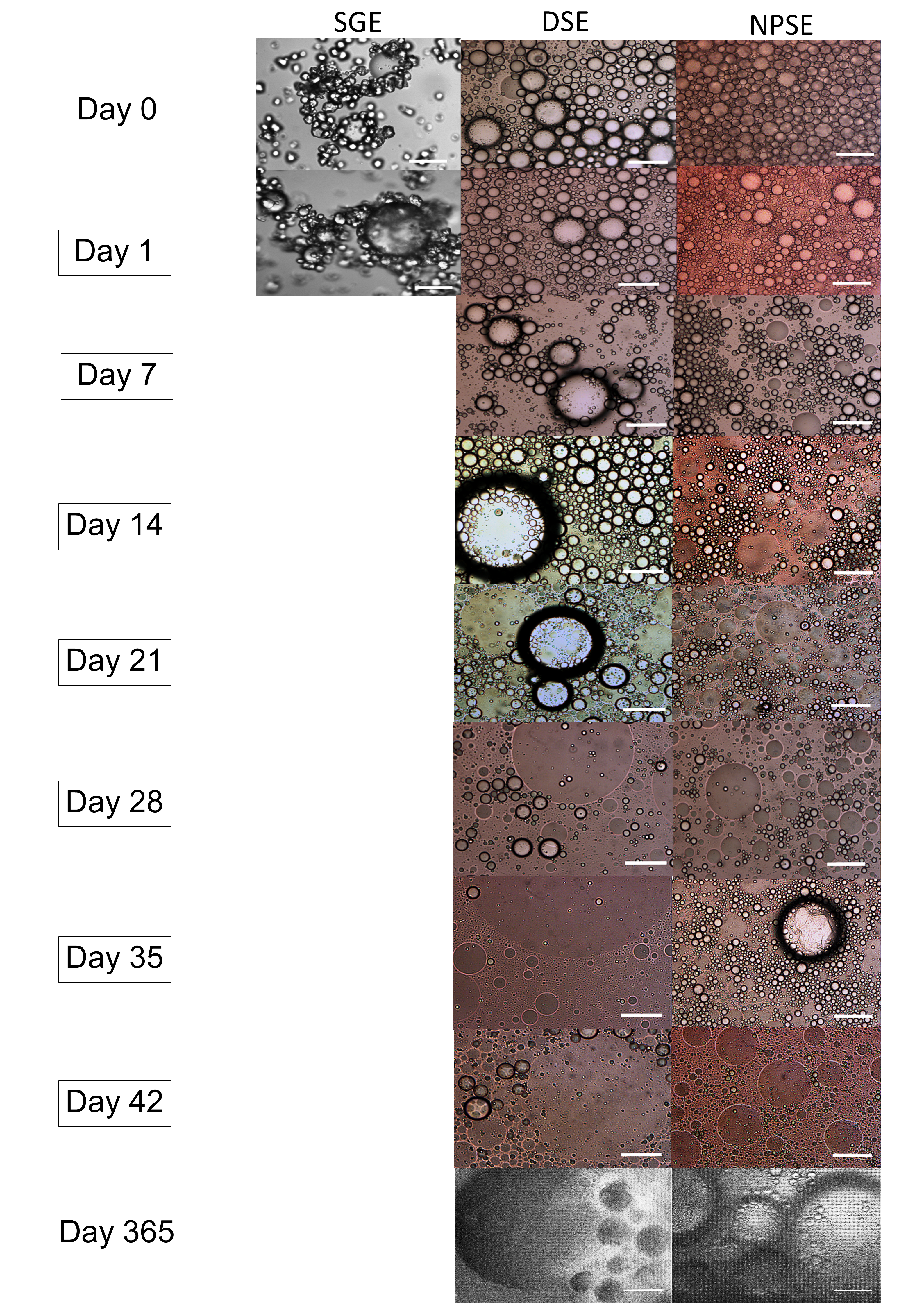

Supplement: S1 Fig — (TIF) [file pone.0210690.s001.tif]
