# Supplementary material for: A comparison of emulsion stability for different OSA-modified waxy maize emulsifiers: Granules, dissolved starch, and non-solvent precipitates
Source: PLoS One. 2019 Feb 6;14(2):e0210690. doi: 10.1371/journal.pone.0210690 (PMC6364883; doi:10.1371/journal.pone.0210690)
Supplement: S1 Table — (DOCX) [file pone.0210690.s002.docx]

| **NPSE (days)** | | | | | | | | | **DSE ((days)** | | | | | | | | **SGE (days)** | | |
| --- | --- | --- | --- | --- | --- | --- | --- | --- | --- | --- | --- | --- | --- | --- | --- | --- | --- | --- | --- |
| **0** | **1** | **7** | **14** | **21** | **28** | **35** | **42** | **365** | **0** | **1** | **7** | **14** | **21** | **28** | **35** | **42** | **0** | | **1** |
| 10.027 | 9.916 | 13.169 | 19.904 | 26.182 | 28.227 | 19.516 | 45.618 | 66.371 | 17.14 | 16.567 | 20.209 | 23.795 | 28.704 | 43.952 | 50.671 | 86.892 | 56.853 | | 81.879 |
| 9.925 | 9.634 | 25.76 | 22.751 | 29.897 | 14.583 | 35.751 | 49.813 | 55.074 | 18.291 | 23.142 | 18.927 | 23.857 | 33.347 | 53.289 | 51.003 | 85.989 | 46.724 | | 86.747 |
| 10.601 | 10.96 | 14.541 | 20.852 | 24.995 | 45.455 | 37.29 | 46.142 | 49.193 | 16.705 | 24.659 | 23.565 | 24.015 | 35.558 | 50.753 | 50.409 |  | | 48.156 | 78.788 |
| 10.686 | 11.237 | 15.263 | 15.046 | 19.501 | 22.92 | 25.34 | 29.963 | 46.117 | 29.657 | 18.124 | 31.246 | 31.026 | 37.188 | 38.99 | 58.752 |  | | 48.971 | 64.951 |
| 11.531 |  | 15.445 | 19.798 | 22.957 | 23.48 | 30.572 | 27.291 | 39.178 | 16.24 | 19.182 | 33.01 | 41.606 | 32.33 | 35.326 | 43.978 |  | | 49.063 | 61.713 |
|  |  | 15.312 | 19.408 | 14.943 | 18.322 | 18.598 |  | 37.632 | 15.501 | 19.496 | 18.911 | 26.531 | 35.814 | 42.02 |  |  | | 34.553 | 70.216 |
|  |  | 14.8 | 18.668 | 18.189 | 16.22 |  |  |  |  |  | 18.559 | 26.807 | 25.574 | 35.557 |  |  | | 35.968 |  |
|  |  |  | 16.391 | 16.962 | 25.218 |  |  |  |  |  | 18.578 | 24.946 |  |  |  |  | |  |  |
|  |  |  |  | 17.336 | 14.676 |  |  |  |  |  |  | 22.382 |  |  |  |  | |  |  |
| **Student T-test** | **0.81701** | **0.00971** | **0.15982** | **0.28354** | **0.58840** | **0.33398** | **0.07066** | **0.18459** |  | **0.62688** | **0.29839** | **0.15480** | **0.05089** | **0.00840** | **0.04522** | **0.00008** | |  |  |

| Comparison at day 0 | **NPSE** | **DSE** | **SGE** | 24 hours (day 1) | **NPSE** | **DSE** | **SGE** |
| --- | --- | --- | --- | --- | --- | --- | --- |
|  | 10.027 | 17.14 | 56.853 |  | 9.916 | 16.567 | 81.879 |
|  | 9.925 | 18.291 | 46.724 |  | 9.634 | 23.142 | 86.747 |
|  | 10.601 | 16.705 | 48.156 |  | 10.96 | 24.659 | 78.788 |
|  | 10.686 | 29.657 | 48.971 |  | 11.237 | 18.124 | 64.951 |
|  | 11.531 | 16.24 | 49.063 |  |  | 19.182 | 61.713 |
|  |  | 15.501 | 34.553 |  |  | 19.496 | 70.216 |
|  |  |  | 35.968 |  |  |  |  |
|  |  |  |  |  |  |  |  |
|  |  |  |  |  |  |  |  |
| **Student t-test** |  | **0.01178596** | **2.0385E-05** |  |  | **0.00033353** | **1.5736E-05** |

**ANOVA (Single Factor)**

| ***NPSE (Days****)* | *Count* | *Sum* | *Average* | *Variance* |
| --- | --- | --- | --- | --- |
| 0 | 5 | 52.77 | 10.554 | 0.411883 |
| 1 | 4 | 41.747 | 10.43675 | 0.60992625 |
| 7 | 7 | 114.29 | 16.3271429 | 17.9027038 |
| 14 | 8 | 152.818 | 19.10225 | 5.9411385 |
| 21 | 9 | 190.962 | 21.218 | 25.2422752 |
| 28 | 9 | 209.101 | 23.2334444 | 93.02517 |
| 35 | 6 | 167.067 | 27.8445 | 64.0607327 |
| 42 | 5 | 198.827 | 39.7654 | 106.89148 |

| *Source of Variation* |  | *SS* | *df* | *MS* | *F* | ***P-value*** | *F crit* |
| --- | --- | --- | --- | --- | --- | --- | --- |
| Between Groups |  | 3256.67885 | 7 | 465.239836 | 11.3381525 | **3.5063E-08** | 2.22122099 |
| Within Groups |  | 1846.49065 | 45 | 41.0331256 |  |  |  |
| Total |  | 5103.1695 | 52 |  |  |  |  |

| ***DSE (days)*** | *Count* | *Sum* | *Average* | *Variance* |
| --- | --- | --- | --- | --- |
| 0 | 6 | 113.534 | 18.9223333 | 28.5250727 |
| 1 | 6 | 121.17 | 20.195 | 9.51566 |
| 7 | 8 | 183.005 | 22.875625 | 35.5382263 |
| 14 | 9 | 244.965 | 27.2183333 | 35.4972045 |
| 21 | 7 | 228.515 | 32.645 | 17.548255 |
| 28 | 7 | 299.887 | 42.841 | 49.6718153 |
| 35 | 5 | 254.813 | 50.9626 | 27.4631313 |
| 42 | 2 | 172.881 | 86.4405 | 0.4077045 |

| *Source of Variation* | *SS* | *df* | *MS* | *F* | ***P-value*** | *F crit* |
| --- | --- | --- | --- | --- | --- | --- |
| Between Groups | 11278.1071 | 7 | 1611.15815 | 54.7246471 | **4.5018E-19** | 2.2370703 |
| Within Groups | 1236.52953 | 42 | 29.4411794 |  |  |  |
| Total | 12514.6366 | 49 |  |  |  |  |

**Comparison between 3 samples at day 0 and 1 (24 hours).**

| *Groups* |  | *Count* | *Sum* | *Average* | *Variance* |  | *Count* | *Sum* | *Average* | *Variance* |
| --- | --- | --- | --- | --- | --- | --- | --- | --- | --- | --- |
| **NPSE** |  | 4 | 42.743 | 10.68575 | 0.43345692 |  | 4 | 41.747 | 10.43675 | 0.60992625 |
| **DSE** | Day 0 | 5 | 96.394 | 19.2788 | 34.7033272 | Day 1 | 6 | 121.17 | 20.195 | 9.51566 |
| **SGE** |  | 6 | 263.435 | 43.9058333 | 45.7485902 |  | 6 | 444.294 | 74.049 | 98.9297228 |

|  | *Source of Variation* | *SS* | *df* | *MS* | *F* | ***P-value*** | *F crit* |
| --- | --- | --- | --- | --- | --- | --- | --- |
| Day 0 | Between Groups | 3077.15385 | 2 | 1538.57693 | 50.0544699 | **1.504E-06** | 3.88529383 |
|  | Within Groups | 368.85663 | 12 | 30.7380525 |  |  |  |
|  | Total | 3446.01048 | 14 |  |  |  |  |
| Day 1 | Between Groups | 12738.1827 | 2 | 6369.09133 | 152.18669 | **9.5592E-10** | 3.80556525 |
|  | Within Groups | 544.056693 | 13 | 41.8505148 |  |  |  |
|  | Total | 13282.2393 | 15 |  |  |  |  |
